# An experimental study on the manufacture and characterization of in-plane fibre-waviness defects in composites

**DOI:** 10.1098/rsos.180082

**Published:** 2018-05-23

**Authors:** W. J. R. Christian, F. A. DiazDelaO, K. Atherton, E. A. Patterson

**Affiliations:** 1 School of Engineering, University of Liverpool, Liverpool, UK; 2 Airbus UK, Filton, UK

**Keywords:** in-plane fibre waviness, composite materials, residual stress, non-destructive evaluation

## Abstract

A new method has been developed for creating localized in-plane fibre waviness in composite coupons and used to create a large batch of specimens. This method could be used by manufacturers to experimentally explore the effect of fibre waviness on composite structures both directly and indirectly to develop and validate computational models. The specimens were assessed using ultrasound, digital image correlation and a novel inspection technique capable of measuring residual strain fields. To explore how the defect affects the performance of composite structures, the specimens were then loaded to failure. Predictions of remnant strength were made using a simple ultrasound damage metric and a new residual strain-based damage metric. The predictions made using residual strain measurements were found to be substantially more effective at characterizing ultimate strength than ultrasound measurements. This suggests that residual strains have a significant effect on the failure of laminates containing fibre waviness and that these strains could be incorporated into computational models to improve their ability to simulate the defect.

## 1. Introduction

Carbon-fibre composite materials are increasingly used for load-bearing aerospace structures as they exhibit high levels of specific strength along the fibre direction. However, if instead of having a desired orientation, the fibre orientation has local variations, the material can be substantially weaker than the design strength. As the shape of the misaligned fibres can usually be approximated as waves, this variation in alignment is termed *fibre waviness*, which can be split into two forms: out-of-plane, where the plies in the laminate are misaligned in the thickness direction; and in-plane, where the fibres within a ply are misaligned [1]. Waviness has the effect of locally reducing both the stiffness and the strength of the plies and, consequently, the limit load for the component. As fibre waviness could threaten the safety of a structure, it is important to explore how such a defect affects the load-bearing characteristics of a structure. There are two routes by which this can be achieved: computer modelling and experimental testing. While a computer model can be informative about the behaviour of a laminate containing waviness, such models often simulate an idealized version of the defect that is not representative of those found in industry [2]. Thus, it is important that experimental studies of fibre waviness are conducted to fully understand its behaviour and to enable validation of computational models. For other composite defects, well-established methods exist for creating typical defects in specimens and thus a substantial quantity of work has been undertaken on the effects of these defects and how they can be characterized. An example of this is impact damage, for which standardized methods of creating the damage have been developed [3], allowing impact-induced damage to be created using a repeatable method. If a similarly repeatable technique existed to create fibre waviness defects in test coupons, then the experimental characterization of waviness could become similarly harmonized.

Out-of-plane waviness has been the subject of a substantial number of experimental studies, in part due to the ease of creating the defect. The out-of-plane deflection of plies is typically created by placing a thin rod at the interface between two plies in a laminate during lay-up [4]. While this results in foreign material being trapped within the cured laminate, the method does produce flat coupons containing waviness suitable for fatigue [4] and fracture studies [5]. In-plane fibre waviness has been the focus of less experimental work, but methods of producing controlled levels of it have been developed. Wisnom & Atkinson [1] used an approximately cylindrical aluminium plate, on which prepreg was laid up to form a laminate. When the lay-up was complete, the plate was flattened causing the upper fibres of the still flexible laminate to buckle, and the laminates were then cured. Other techniques for producing in-plane waviness have been demonstrated, including: jetting gas through dry fibres prior to resin impregnation, and thermally buckling fibres [6]. These methods result in flat coupons with the in-plane waviness distributed uniformly throughout the composite and thus are not consistent with waviness defects observed in real components which are typically localized [2]. Localized waviness has been produced using prepreg plies that are oversized for the laminate that contains them; when the plies are forced into a smaller area than their actual size, the fibres buckle, resulting in a combination of out-of-plane and in-plane waviness [7]. Other techniques have focused on producing specific shapes of waviness by manipulating dry fibres into waves [8] and crimping plies before flattening the ridges into in-plane waves [9]. The reverse of the technique in [1] has been used to produce local waviness at an L-bend in a composite component [10], i.e. a flat laminate was laid up that was then pressed into an L-bend mould causing the fibres on the inner radius to buckle. This method forms localized waviness; however, it is difficult to vary the size and severity of waviness and it results in complex-shaped specimens which are difficult to characterize. However, if the laminate was instead laid up over a former with a specific shape, then the shape of the former could be varied to achieve different positions, size and severity for the waviness defect. This is the approach that has been adopted in this study.

Once fibre-waviness specimens have been produced, it then becomes necessary to characterize the defect. Previous research on the characterization of fibre waviness, both in-plane and out-of-plane, has focused on measuring the orientation of fibres within a laminate. Fibre orientation can be measured destructively by sectioning the laminate [11] or non-destructively with ultrasound [12], radiography [13] and eddy-current [8] techniques. Ultrasound is perhaps the most promising inspection method due to it already being a common method of non-destructively evaluating composites. While many techniques have been investigated for their potential for inspecting fibre waviness, there have been no reported attempts to directly predict the strength of the laminates from measurements of fibre waviness using empirical models; nor any exploration of whether measurements of fibre orientation are the most effective non-destructive technique with which to characterize a laminate. An alternative approach to detecting waviness defects is to use non-contacting strain measurements to locate and characterize areas of reduced stiffness in the composite. Out-of-plane defects can be detected using thermoelastic stress analysis [14], but no attempt has been made to determine the severity of the defects and their effect on remnant strength using strain measurements. Here, strain-based non-destructive inspections are used to characterize laminates containing waviness with a methodology similar to previous work on impact-induced damage [15].

This paper introduces a method to allow for the creation of localized areas of in-plane fibre waviness. A large number of specimens were produced and then characterized using: ultrasound, digital image correlation (DIC) strain measurements and optical shape measurements. The fabrication and characterization techniques are described in §2 and the results presented in §3. The results are discussed in §4 and concluding statements made in §5.

## 2. Experimental method

### 2.1. Specimen fabrication

**Figure 1.**
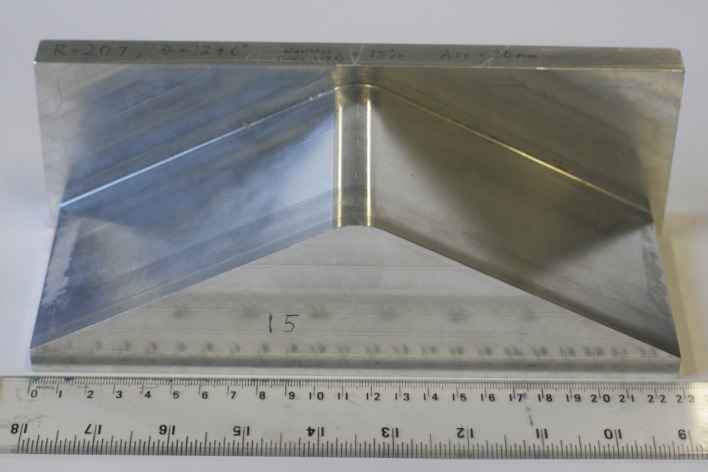
Photograph of the former used for creating specimens with a nominal waviness of 15%.

Quasi-isotropic laminates were produced with six levels of waviness severity. Six specimens were produced at each level to explore the variability of the defect generation technique. This resulted in a large set of 36 specimens. The laminates were manufactured using RP507UT210 (PRF, UK) unidirectional prepreg with a [0_2_/90_2_/45_2_/-45_2_/-45_2_/45_2_/90_2_/0_2_] lay-up with no debulking during lay-up. This resulted in symmetric laminates consisting of eight plies, with an average ply thickness of 0.37 mm after curing. Each level of waviness was produced using a milled aluminium former. The profile of each former had an arc at its centre from which flat surfaces extended on either side; a photograph of one former is shown in figure 1. Prepreg plies were laid up over each former by hand so that the plies closest to the arc surface of the former have a shorter path than the plies further from the arc surface. Once the prepreg laminate was laid up, it was removed from the former and flattened between two plates using only minimal force provided by hand. In this uncured state the laminates had a nominal thickness, $t_{u}$, of 3.65 mm. When the prepreg laminates were flattened, the fibres in the plies on the top surface buckled to accommodate the constraint within the flattened laminate. These buckled fibres are the cause of the in-plane fibre waviness. The bottom surface of each specimen was visually inspected to ensure that it was free of waviness; and no waviness was observed on the bottom of any specimens. The nominal waviness in the uncured laminates produced by each former was quantified by the percentage reduction in length of the top of the laminate over the curved section, $l_{\mathrm{top}},$ relative to the length of the same section after flattening. The length after flattening is expected to be similar to the length of the bottom of the curved section prior to flattening, *l*_bottom_. This is because the stiffness of the uncured plies in the fibre direction is much higher in tension, where the stiffness of the fibres dominates, than it is in compression, where the uncured resin allows the fibres to buckle, resulting in the neutral axis being close to the bottom surface of the laminate. The nominal waviness is therefore equivalent to the percentage excess length of the fibres in the top ply and can be expressed as follows:

2.1 $$\text{Nominal waviness}=\frac{l_{\mathrm{top}}-l_{\mathrm{bottom}}}{l_{\mathrm{top}}},$$

where

2.2 $$l_{\mathrm{top}}=\frac{\pi \theta }{180}(r+t_{u})$$

and

2.3 $$l_{\mathrm{bottom}}=\frac{\pi \theta }{180}r$$

and where *r* is the radius of the arc section of the former, $t_{u}$ is the thickness of the uncured laminate and *θ* is the arc central angle. By substituting equations (2.2) and (2.3) into (2.1), the nominal waviness can be expressed in terms of the radius of the former and the thickness of the uncured laminate as follows:

2.4 $$\text{Nominal waviness}=\frac{t_{u}}{r+t_{u}}.$$

As the thickness of the uncured laminate is typically the same for every specimen, the nominal waviness can be varied by changing the arc radius of the former. The nominal length of the waviness defect, which is equal to the length of the bottom of the curved section of the unflattened laminate, *l*_bottom_, can also be varied by changing the central angle using equation (2.3). The nominal length of the defects after flattening was kept constant at 20 mm. The six levels of nominal waviness chosen were: 0%, 10%, 15%, 17.5%, 20% and 25%. The arc radii and arc central angles for the corresponding five formers are listed in table 1.

**Table 1.** Key dimensions of the formers to obtain given levels of nominal waviness over a 20 mm length of coupon. The arc central angle is the angle swept by the radius from each end of the arc; an arc central angle of 180° would therefore be a semicircle.

nominal waviness (%)	arc radius, *r* (mm)	arc central angle, *θ* (°)
10.0	32.9	34.9
15.0	20.7	55.4
17.5	17.2	66.6
20.0	14.6	78.5
25.0	11.0	104.6

**Figure 2.**
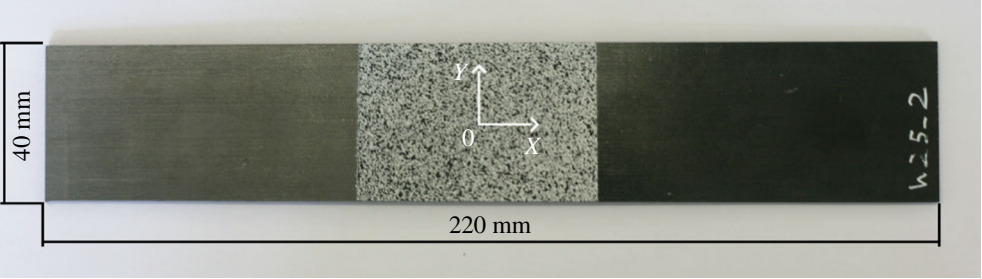
Photograph of a specimen with a nominal waviness of 25%, showing the speckle pattern, dimensions and the origin for the measurement coordinate system at the centre of the speckle pattern.

Then, the laminates were cured between flat plates in a hot press (APV-3530, Meyer, Germany) according to the manufacturer's instructions. The rate of heat and pressure application was controlled by the machine and thus did not vary between specimens. The plates were heated at a rate of 10°C min^−1^ to a temperature of 130°C while being subjected to 2.5 bar pressure, and were held at this temperature and pressure for 45 min. Once the laminate was cured, the hot press was left to cool to room temperature over 6 h before the pressure was released and the specimen removed. The laminates were then cut into 40 mm by 220 mm coupons using a wet diamond saw (Versatile 103450, Vitrex, USA). Care was taken to ensure the long edge of the specimens was parallel to the 0° direction of the fibres and that the wavy area was close to the specimen centre. This resulted in the 0° fibres in the top and bottom plies running parallel to the *X*-direction, as indicated in figure 2.

To examine the effect of high levels of waviness, two specimens were produced with the top 0° plies replaced with 90° plies. This corresponds to the worst possible case of fibre waviness, as it represents the complete misalignment of fibres and thus was used to determine how much the ultimate bending moment can be reduced due to fibre waviness. These specimens were cured using the same process described previously and cut to the same size coupons as the other specimens.

Additional waviness specimens were produced with a nominal waviness of 0% and 20%. These were sectioned in order to perform microscopy on: the cut edge running in the *X*-direction, to observe out-of-plane waviness; and the defective ply surface, to observe in-plane waviness. The sectioned specimens were mounted in epoxy and then polished until the individual fibres could be observed. Images were then captured using an inverted microscope (Epiphot, Nikon, Japan).

### 2.2. Ultrasonic characterization

**Figure 3.**
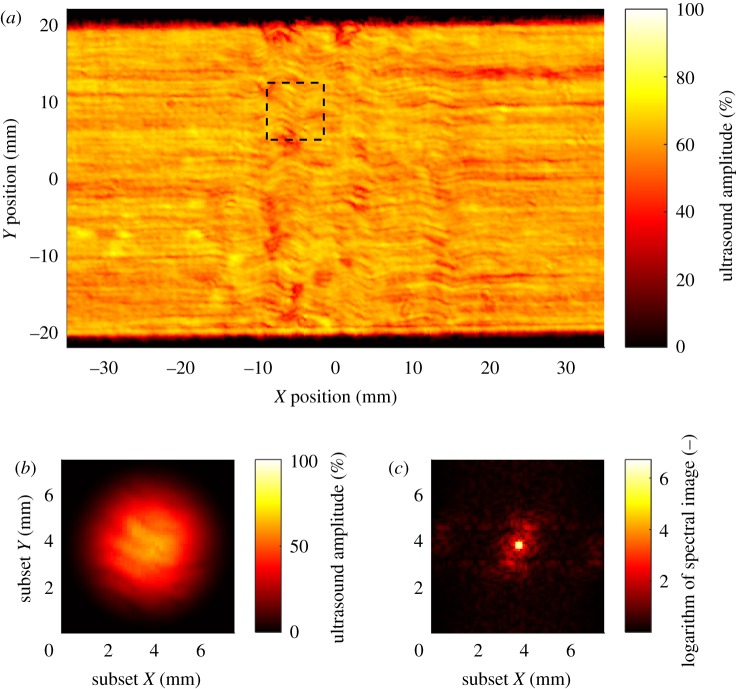
An amplitude C-scan of a 17.5% nominal waviness coupon showing the waviness in the top ply (*a*) with a subset of ultrasound data indicated by a dashed rectangle. A Hann window was applied to the subset (*b*) and its spectral image (*c*) used to determine the fibre orientation at the subset location.

Each specimen was inspected using immersion pulse-echo ultrasound to measure the fibre orientation. A multi-axis scanner (Midas NDT, UK) was used to produce C-scan amplitude images of the interface immediately below the 0° ply on the top surface of the laminate. The scanner had a focused probe with a natural frequency of 10 MHz, and the probe and coupon were immersed in water for ultrasonic coupling. The probe was attached to an ultrasonic flaw detector (Epoch 4+, Olympus, Japan) that produced an A-scan of the laminate at the location of the probe. The amplitude in a gated portion of the A-scan, corresponding to the echoes from a 0.1 mm thick layer of the laminate at the first ply interface, was recorded. The echo amplitude was recorded at 100 µm increments along lines with a spacing of 200 µm, resulting in an amplitude C-scan. The fibre bundles in the 0° ply were visible in the texture within the C-scans, and thus the local orientation of the fibres could be measured. This was achieved by performing the two-dimensional discrete Fourier transform on square subsets of the C-scan. The size of these subsets was 75 pixels (7.5 mm) and the subsets had a spacing of 10 pixels (1 mm), resulting in a grid of orientation measurements with a pitch of 1 mm across the surface of the specimen. Prior to calculating the Fourier transform, a radial Hann window was applied; this limits spectral leakage, which would cause the peak at the centre of the spectral image to broaden [12]. A windowed subset is shown in figure 3*a*. The power spectrum obtained had a roughly elliptical shape at its centre with its major axis in the transverse fibre direction, as shown in figure 3*b*,*c*. Thus, by measuring the orientation of the power spectrum ellipse, the local fibre orientation was obtained. This technique, of using ultrasound to measure fibre orientation, is described in detail in [12].

### 2.3. Digital image correlation characterization

DIC was used to measure strain on the surface of each coupon as it was loaded to failure. A speckle pattern was applied to a 60 mm by 40 mm section of each coupon at the location of the fibre-waviness defect on the opposite surface to the defect. A white base coat (Matt White, PlastiKote, USA) was applied to the specimen and then black speckles (Pro Paint Acrylic Black Matt, CRC, USA) sprayed over the top. This resulted in speckles with a nominal diameter of 0.25 mm. A sprayed specimen is shown in figure 2. Each coupon was loaded to failure in bending using a servo-hydraulic load machine (8501, Instron, USA). The specimens were held in a four-point bend rig with a 160 mm support span and 80 mm load span. Fibre waviness has been shown to have the greatest effect on compressive strength [16,17] and thus the defective ply was placed into compression when loaded. Stereoscopic DIC (Q-400, Dantec Dynamics, Germany) was performed using a pair of cameras facing the specimen from either side of the four-point bend rig. The DIC analysis was performed using a facet size of 25 pixels and grid spacing of 5 pixels. Strain was calculated by differentiating quadratic surfaces fitted to the DIC displacement data [18]. Each surface was obtained by taking square subsets of the displacement field that contained 441 displacement vectors and fitting a polynomial surface using the method of least squares.

For the tests, each specimen was initially rested on the lower pair of load noses of the bending rig. The reference images for DIC were captured at this stage so that the unloaded curvature of the specimens was measured. The lower half of the bending rig was then moved towards the upper load noses at a constant displacement rate of 0.8 mm min^−1^. The specimens were loaded with the fibre-waviness defect on the top surface of the laminate in compression, and the lower surface, on which the speckle pattern was applied, in tension. The DIC system was set to capture images every 15 s; this resulted in a bending-rig displacement of 0.2 mm between each image. The test was stopped when the stiffness of the coupon was reduced to approximately half its initial value.

## 3. Results

**Figure 4.**
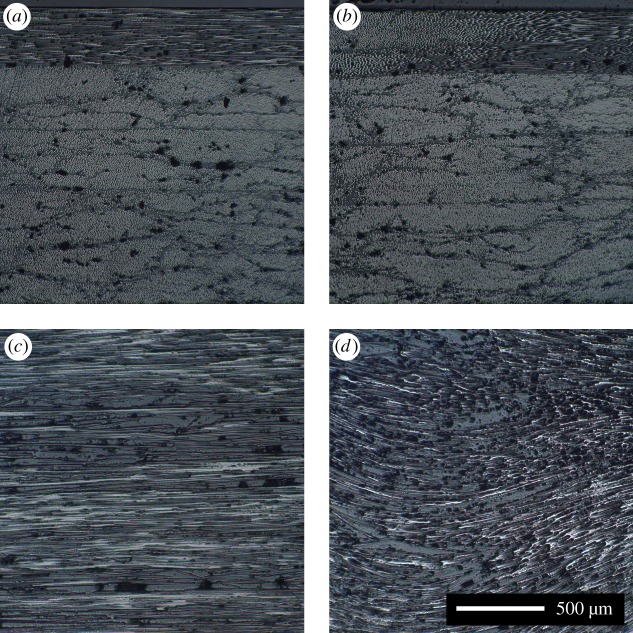
Microscope images of the fibres in a 0% nominal waviness specimen (*a*,*c*) and a 20% nominal waviness specimen (*b*,*d*), showing for each specimen its edge (*a*,*b*) and its top surface (c,*d*).

The waviness was observed in the two sectioned specimens using microscopy. The polished edge and ply surface of a 0% waviness specimen are shown in the two images in figure 4*a*,*c*. The two images in figure 4*b*,*d* are of the same features but for a 20% nominal waviness specimen. After the images in figure 4 were captured, the mounted specimen sections used for the bottom pair of images were further ground to reveal the 90° plies beneath the top plies. These were then repolished and visually compared using microscopy to qualitatively determine that in-plane waviness was not present in the 90° plies.

**Figure 5.**
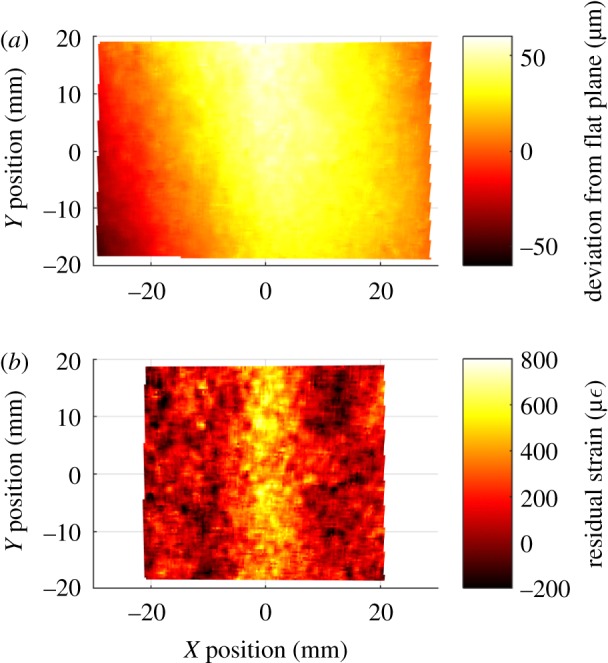
DIC measurements of the surface deviation from a flat plane for an unloaded specimen with a nominal waviness of 17.5% (*a*) and the associated residual strain field (*b*).

After the specimens were cured and cut into coupons, it was observed that despite being cured between flat plates, the specimens were slightly curved along their length in the *X*-direction. This curvature was caused by residual strains in the laminate. As the curvature of the specimens was small, the residual strains in the specimens that caused the curvature were determined from the out-of-plane displacements [19]. The residual strains in the direction along the length of the specimen were calculated as follows:

3.1 $$\epsilon_{x,\mathrm{res}}=-\frac{t_{c}}{2}\frac{\partial^{2}w}{\partial x^{2}},$$

where *t*_c_ is the cured thickness of the laminate and *w* is the displacement of the defect-free surface from a flat plane. For each specimen, the DIC reference image was processed to measure the shape before any loads were applied, resulting in the data shown in figure 5*a*. The second partial derivative of these shape data was then calculated using the central difference method [19] with a step size of 8 mm, resulting in the residual strain field shown in figure 5*b*.

**Figure 6.**
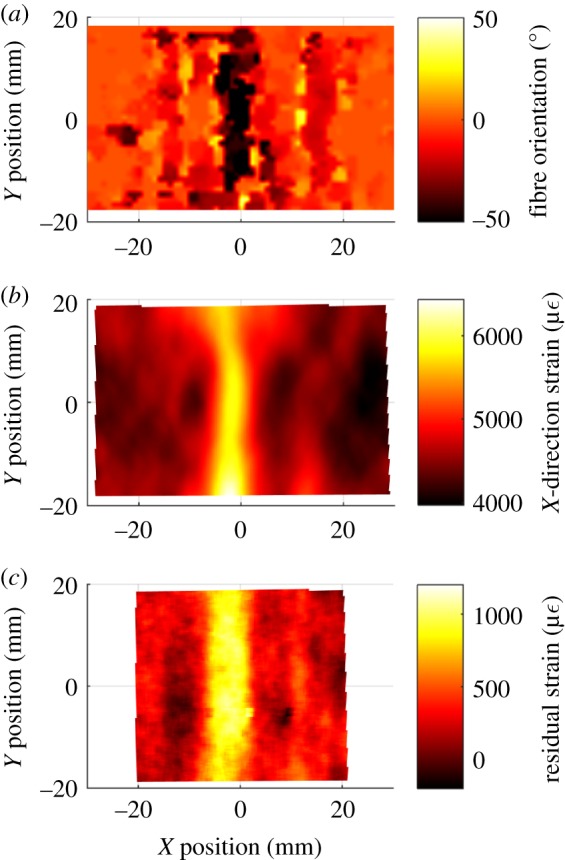
Data from a specimen that had a nominal waviness of 25%, inspected with: ultrasound (*a*), surface strain at a load of 22 Nm (*b*) and residual strain measurements (*c*).

The fibre waviness was apparent in the ultrasound data, in the DIC strain data when the specimens were loaded, and also in the residual strain maps when no load was present. It was also possible to identify the same spatial distribution of the waviness defect in each specimen using the three distinct inspection techniques. Data from a specimen that had a nominal waviness of 25% are shown in figure 6. A strip of highly non-aligned fibres, with fibre angles around −50°, is visible across the width of the specimen, at *x* = −2 mm in the ultrasound data, and a corresponding area of high strain is visible in the strain field and the residual strain field. In the ultrasound data, there is not a corresponding strip of fibres orientated at +50°; but only small areas of fibres orientated at about +25° on either side at around *x* = −10 mm and *x* = +10 mm. This perhaps implies that the waviness is z-shaped, which would be consistent with other studies where complicated shapes have been observed [20]. A less severe strip of waviness is also visible in all three datasets, at *x* = 17 mm.

**Figure 7.**
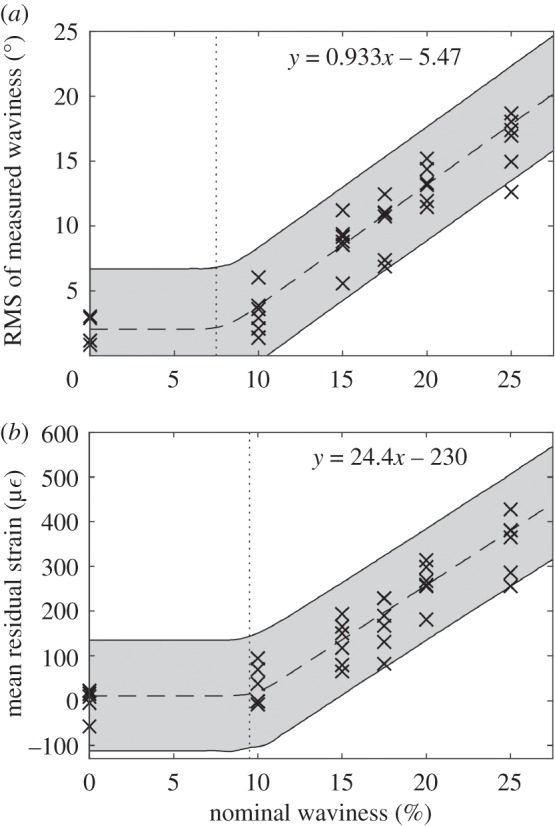
The ultrasound-measured waviness (*a*) and mean residual strain (*b*) for specimens after curing for six different levels of nominal waviness, with the 95% credible interval shown in grey, the threshold of detectable waviness indicated by a dotted line and the equation for the relationship above the threshold shown at the top of each graph.

The overall waviness measured using ultrasound was quantified by calculating the root mean square (RMS) value of the fibre orientation fields. By calculating the RMS value of the ultrasound data, both local misalignment due to the waviness defect and any gross misalignment of the plies were quantified. When waviness was present, a correlation between the nominal waviness and the RMS of the measured waviness was observed and is shown in figure 7. Piecewise robust Bayesian regression [21] was used to determine a function linking the nominal waviness to the measured waviness after curing. Bayesian regression was used for several reasons. Firstly, because it allows the incorporation of prior knowledge, which might come from expert knowledge or physical intuition. Secondly, it allows the computation of credible intervals that attach a measure of uncertainty to predictions. Thirdly, because it provides a coherent treatment of possible outliers in the data. The Bayesian regression model employed in this work is similar to that used in [22] but with an additional parameter that controls the point at which the slope of the regression line transitions from zero to a non-zero value. The prior information about this transition parameter was modelled as having a uniform prior distribution across the entire range of nominal waviness, indicating that the transition is equally likely to occur at any value of nominal waviness. The regression model was fitted using Gibbs sampling [21], performed using the software package JAGS [23]. The grey region shown in figure 7 is the 95% credible interval and indicates the most probable range of the measured waviness after curing for a given nominal waviness.

**Figure 8.**
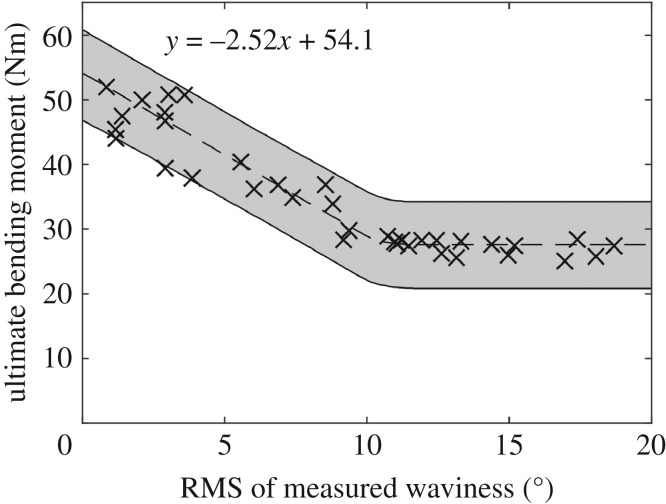
The effect of waviness after curing on the ultimate bending moment for all specimens, with the 95% credible interval shown in grey and the equation for the line-of-best-fit when measured waviness is less than 10° shown at the top.

**Figure 9.**
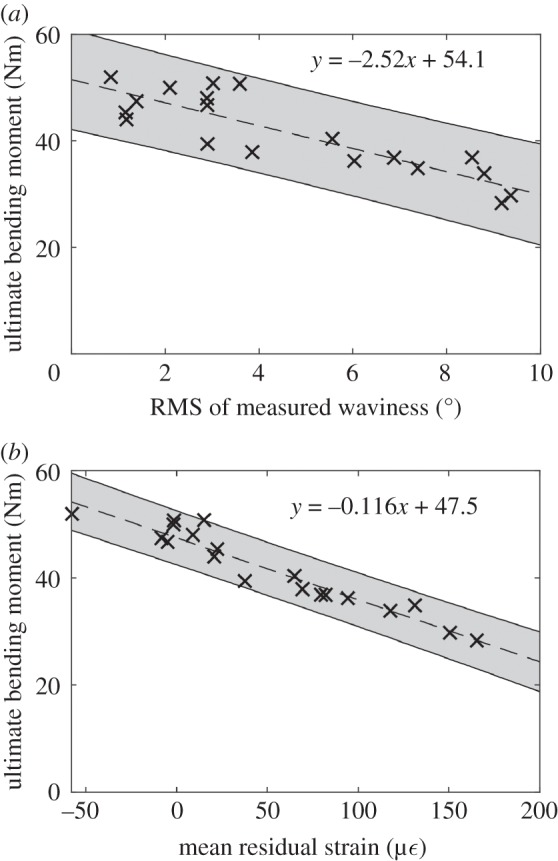
Predictions of the ultimate bending moment of specimens using RMS values of waviness measured with ultrasound (*a*) and the mean of the residual strain field (*b*), with the 95% credible interval shown in grey and the equation for the line-of-best-fit shown at the top of each graph.

The ultimate bending moment of the specimens containing fibre waviness was also found to correlate with the RMS of the measured waviness, as shown in figure 8. A linear correlation between the measured waviness and ultimate bending moment was observed when the measured waviness was less than 10°. When the measured waviness was above 10°, the specimens failed with an average ultimate bending moment of 27.7 Nm, regardless of the measured value of waviness. The two specimens with the top 0° plies replaced by 90° plies were loaded to failure and found to fail at 30.3 and 29.4 Nm. These specimens had an RMS of measured waviness of 90°. The effectiveness of using ultrasound measurements of waviness to predict the strength of laminates was then explored. Specimens with an RMS of waviness below 10° were used to fit two robust Bayesian linear regression models, as described in [22]. The input for the regression models were: the RMS of the ultrasound-measured waviness, and the mean of the residual strain field. It was clear from the graphs of the two fitted regression models, in figure 9, that both measurements have a strong linear correlation with the ultimate bending moment. The average prediction uncertainty of the two regression models was estimated using leave-one-out cross-validation (LOOCV) [24]. The average prediction uncertainty was 3.94 Nm for ultrasound-based predictions and 2.24 Nm for predictions based on residual strain measurements.

**Figure 10.**
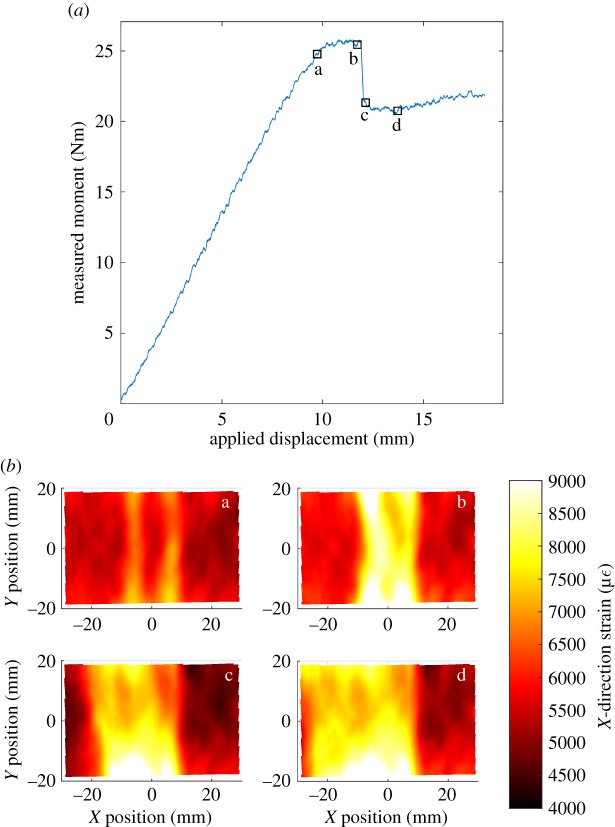
Load–displacement graph for a specimen that had a nominal waviness of 25% loaded to failure with four values on the load curve marked (*a*) and the strain fields for these values (*b*).

**Figure 11.**
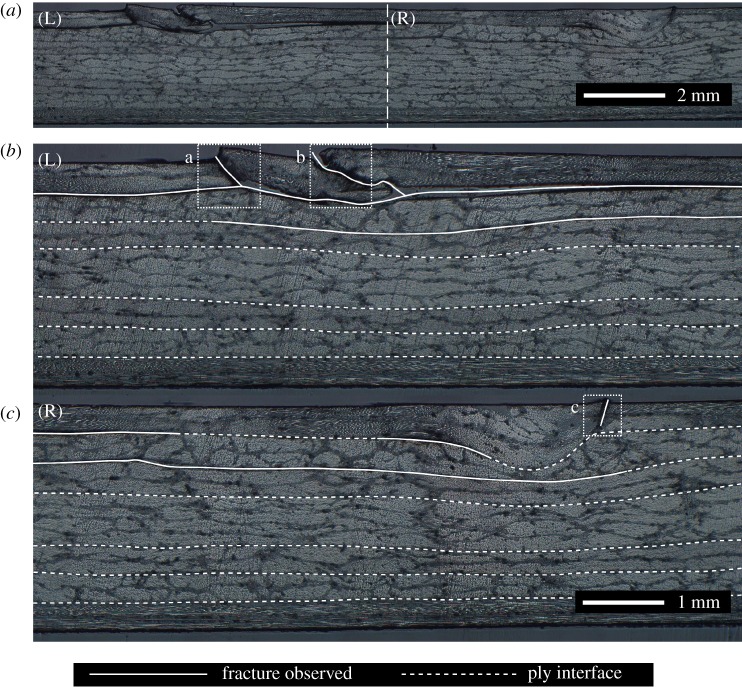
A microscope image (*a*) of the same 25% nominal waviness specimen shown in figure 10 after loading to failure, with enlarged views of the left (*b*) and right (*c*) portions with solid lines indicating observable fractures, while the dashed lines indicate the ply interfaces. The top defective ply was fractured at three locations (indicated by dotted rectangles) and higher magnification views of these locations are provided in figure 12.

**Figure 12.**
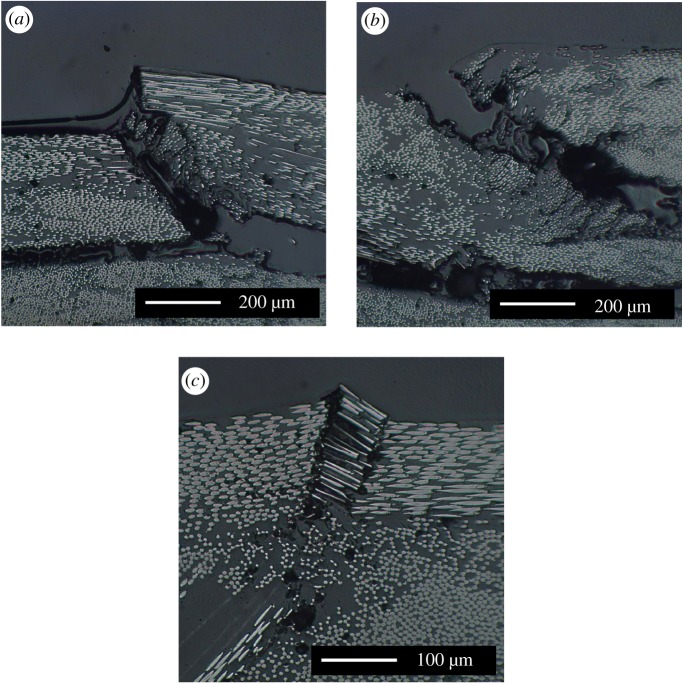
High-magnification images of the fractures through the top defective plies. The letter in the top left corner of each image references the locations marked in the low-magnification image in figure 11.

Strain fields on the surface of the fibre-waviness specimens were captured while loading the specimens to failure. These strain fields allowed the progression towards failure of the fibre-waviness defects to be observed. The load–displacement curve for a specimen that had a nominal waviness of 25% is shown in figure 10, and, for four different points, indicated by square markers, the corresponding strain fields are also shown. The first strain field was captured at a subcritical load, just prior to the start of damage propagation; the subsequent three strain fields show the damage during the propagation process. This specimen was also sectioned such that the edge of the specimen close to the section containing waviness could be observed using microscopy; this is shown in figure 11. The edges of the delaminations formed during loading that could be visually identified using microscopy have been marked using solid white lines. Fractures through the top defective ply were also observed; these are marked using dotted rectangles. In the left side of the micrograph, these fractures (at ‘a’ and ‘b') have caused severe deformation of the microstructure so that the waviness from which they originated is no longer apparent. However, the fracture ‘c' shown in the right micrograph is less severe and the waviness defect is still apparent in the microstructure. High-magnification images for the three fractures are shown in figure 12.

## 4. Discussion

Waviness defects in composite laminates can be caused by: misalignments in the unidirectional prepreg tape used to produce the laminate; the geometry of the tooling for the desired component shape; or the orientation of the reinforcement fibres [25]. Thermal stresses produced during the curing process can also result in waviness defects [26]. The fibre-waviness defects in this study were produced by inducing axial strain in the top surface of the uncured laminate causing the fibres to buckle. The authors accept that this is not a typical process by which fibre-waviness defects occur in industry, but it does result in defects that are suitable analogues for the study of both the mechanical behaviour of the defects and the non-destructive evaluation techniques used to detect them. The specimens produced in this study have a constant thickness across their surface due to the rigid platens of the hot press; whereas aerospace components are typically manufactured using autoclaves and thus slight variations in thickness might occur at the defect locations, which could be created in laboratory specimens by using an autoclave instead of a hot press.

Some waviness is always present in the prepreg prior to lay-up [11]; as all of the specimens were obtained from the same batch of material, this initial level of waviness was constant and thus variations in the measured waviness were solely due to the formers. The plies in this study were 0.37 mm thick, which is greater than those found in composite structures, which typically have ply thicknesses of 0.125 mm [27]. This helped to increase the amplitude of the ultrasound echoes and thus the contrast in the C-scan images. An example of the waviness produced in a specimen with a nominal waviness of 20% is shown in figure 4. While slight out-of-plane waviness can be observed in figure 4*b*, the deflection of the plies from the horizontal is minor compared to the in-plane waviness observed in figure 4*d*. Also, when the top ply was removed, the 90° ply beneath it was found to have no discernible level of in-plane waviness. These two observations suggest that the defect is only significant in the top 0° ply and that the rest of the specimen can be assumed unaffected by the process of creating the defect. Voids can be observed in the micrographs in figure 4 as small black shapes distributed throughout the four images and are probably responsible for some of the scatter in the results presented in figures 7–9; nevertheless, the large number of samples investigated allows conclusions to be drawn. As the volume of voids in a composite is related to the pressure used during curing, the use of higher pressures would probably have resulted in a lower volume of voids [28].

Three distinct techniques have been used to inspect the specimens. The first technique was the use of ultrasound to measure the fibre orientation in the top ply of the specimens, allowing the defects to be located and their severity quantified. While these ultrasound measurements provide information on the general orientation of the fibres, it is difficult to directly determine the shape of the buckled fibres due to the complex shape of the fibres and the limited spatial resolution of the measurement technique [29]. The second technique used DIC to measure the strain redistribution due to the waviness defect. Finally, the curvature of the specimens was used to locate defects. The curvature was caused by residual stresses in the laminate at the defect location. These residual stresses are due to the mismatch of thermal expansion coefficients between the laminate layers because of the orthotropy of the unidirectional plies [30]. If the laminate lay-up is symmetric, the stresses produced during cooling are balanced, resulting in a flat laminate. For this study, the laminates had a symmetric quasi-isotropic lay-up and thus the residual stresses should be balanced; however, at the waviness defect, the fibre misalignment results in localized areas where the residual stresses are not balanced. These unbalanced residual stresses and the associated residual strains result in a slight curvature at the location of the defect. By calculating the second partial derivative of the out-of-plane displacement, these unbalanced residual strains can be evaluated and the residual strain fields obtained, as shown in figures 5*b* and 6*c*. While the measured residual strains are significant, they are still small relative to the surface strains that can be reached when loading is applied. For example, on the 0% nominal waviness specimens, strains in excess of 10 000 µε were measured at ultimate load, while the largest residual strains measured on any of the specimens containing waviness was approximately 1200 µε. The shape data used for calculating the residual strain fields were measured by the same DIC system used to calculate surface strain in the loaded specimens. While the raw data for these two measurements were obtained from the same source, the processing of the data is completely different and similar measurements of residual strain would be obtained by any suitably sensitive shape measurement technique. Similarities in the spatial distribution of the defects detected by the three inspection techniques were observed for all specimens with significant fibre waviness.

A relationship was found between the nominal waviness and the ultrasound-measured waviness after the specimens were cured. The nominal waviness is a quantity derived from the shape of the formers used to create the specimens and should not be confused with the ultrasound-measured waviness which is a measurement of the actual waviness present after the specimens were cured. When the nominal waviness was over 10%, a linear correlation was observed between the nominal and measured waviness, with the data points being widely spread above and below the line-of-best-fit; this is shown in figure 7. The spread of data is due to the stochastic process by which the fibres buckle and thus form the defect. Between 0 and 10% nominal waviness, this correlation was not observed which was probably due to the noise floor of the ultrasound measurements, preventing detection of the defects. By using piecewise robust Bayesian regression, the minimum nominal waviness that would be expected to cause a measurable change in the fibre orientation was estimated to be approximately 7%. This level of nominal waviness could be used to produce specimens with a level of waviness that is at the threshold of detectability. These specimens could then be loaded to failure to obtain conservative estimates of ultimate strength for designing structures that may contain undetectable fibre waviness. A similar relationship was observed between nominal waviness and mean residual strain and is shown in figure 7*b*.

A correlation is evident in figure 8 between the RMS of the measured fibre waviness and the ultimate bending moment for the specimens. For low levels of measured fibre waviness, a linear relation exists between the measured waviness and residual strength. At higher levels of waviness, this linear correlation was no longer present and the specimens failed with a mean value of 27.7 Nm. It is likely that this behaviour arises because the stiffness in the gross fibre direction of the 0° ply containing the defect cannot be reduced to less than the transverse stiffness of the ply which still has some limited load-carrying capacity. This was explored using the two specimens produced with the top 0° ply replaced with a 90° ply. The two specimens failed at an average bending moment of 29.9 Nm, which is 8% higher than the average failure load for severe fibre waviness. The lower failure load in the presence of in-plane fibre waviness could be due to minor out-of-plane waviness, such as that observed in figure 4*b*, as well as the fibre-waviness defect acting as a stress concentrator, causing higher stresses at the defect than if the stiffness of the ply was uniformly reduced. It is likely that in-plane waviness in other plies had no influence, as waviness was not observed in the lower plies of the sectioned 20% nominal waviness specimen.

The correlation between the ultrasound measurements and ultimate bending moment implies that strength predictions can be made using these measurements, as shown in figure 9. The accuracy of these predictions was quantified only for specimens containing measured waviness up to an RMS value of 10° as the relation was linear in this range. The mean of the measured residual strain field was also used as a predictor to examine whether the residual strains produced by waviness are important for predicting the ultimate strength. One specimen, with a nominal waviness of 0%, was observed to have a mean residual strain of −58 µε. This specimen had the highest ultimate bending moment of all the tested specimens. The negative mean residual strain could have been caused by a thermal gradient through the thickness of the specimen during curing. As the regression model was robust against outliers [21], the data from this specimen were not omitted. When the LOOCV performance metric was calculated for the two regression models shown in figure 9, it was found that the predictions obtained using residual strain measurements had an uncertainty that was only 57% of that for ultrasound-based predictions. While the ultrasonic non-destructive evaluation being developed in industry focuses on measuring the size and shape of defects, the intention of these inspections is still to assess whether the performance of a structure will be degraded by the defect. The residual strain-based assessment in this study still achieves this end goal, but the predictions are significantly more accurate. Residual strains are known to affect the remnant strengths of laminates [31] and thus the high correlation is probably due to this effect. Furthermore, given the high levels of strain measured and the strong correlation between residual strain and failure load, the correlation suggests computational models that attempt to predict the strength of laminates containing fibre waviness could be improved by incorporating the residual stresses at the location of the defect. It also implies that residual strains obtained from optical shape measurements could be used to locate and quantify the severity of waviness defects, provided that the laminate has a constant thickness. This would be difficult to perform for components with complicated shapes, as the shape when residual strains are not present must be known, but may be possible on planar sections of components as well as cylindrical shells. This would potentially be simpler for load-bearing structures, which have more basic shapes than aerodynamic structures such as the outer surfaces of wings. The technique would be unable to detect defects that are on the mid-plane of a specimen as it would still be symmetric and thus measurable residual strains would not form during curing. However, for the fibres to only buckle at the mid-plane the uncured specimen would have to experience compressive stresses at the mid-plane while on either side the specimen was unstressed or in tension. It is unlikely that this stress state would occur and thus laminates containing fibre waviness are expected to be asymmetric. These predictions of ultimate bending strength could still be obtained for curved components using ultrasound, as proprietary systems exist specifically for conducting ultrasonic C-scans on curved components such as aerofoils and pipes. Additional inspection time may be required to obtain C-scans of each potentially defective ply. However, once the ultrasonic C-scan data are obtained, they can be converted to fibre orientation measurements using the same algorithm described in §2.2, which is based on common signal processing techniques and would require minimal input from the inspecting technician.

Finally, DIC has been used to observe the progression towards failure of all the specimens. A typical example of these data for a specimen with a nominal waviness of 25% is shown in figure 10. At a subcritical load, strain field ‘a' showed two strips of approximately equal levels of strain running parallel to the *y*-axis at *x* = −7 mm and *x* = +7 mm. After strain field ‘a' was captured, cracks were observed forming through the 0° ply on the top surface at *x* = −7 mm, running parallel to the *y*-direction. When this specimen was inspected using microscopy, as shown in figure 11, these cracks were found to run from the top surface of the specimen down to the first ply interface, marked at the top of figure 11 with high-magnification images in figure 12. The formation of these cracks corresponded with the end of the linear relation between bending displacement and measured moment, confirming that damage was being created. A substantial strain concentration was visible in strain field ‘b' at the location of these cracks. When the applied bending displacement reached 12 mm, a sudden drop in the stiffness occurred, which corresponded with a delamination that formed and immediately buckled. The effect of this delamination can also be observed in strain field ‘c' in figure 10 with a large area of high strain running between *x* = −18 mm and *x* = 8 mm. This area of high strain increased in size by growing further in the negative *x*-direction as the bending displacement was increased, as can be seen in strain field ‘d' in figure 10. The delamination was also observed in the microscope images, running off the left side of figure 11. An additional delamination was also observed at the interface between the 90° and 45° plies. This second delamination was only present in the waviness area and was probably formed after the main delamination, as the strains close to the mid-plane of a specimen under bending are lower than those at interfaces further away. This progressive failure, whereby a crack forms through the 0° ply at the defect location, from which a delamination grows, was observed in all of the specimens with a mean residual strain over 40 µε and an RMS value of fibre waviness over 3.9°.

By testing specimens across a wide range of nominal waviness levels, this study has shown that in-plane fibre waviness can result in significant reductions in the load-bearing strength of laminates. If similar defects occurred in an aerospace structure, this could result in the structure failing before it reached its design ultimate load. The shape of the buckled fibres that form the waviness defect, and the way the defect affects remnant strength is subject to high levels of variability due to the complex microstructure of the heterogeneous composite material. The technique for generating fibre waviness developed in this study allows the variability in the shape and severity of the waviness defects to be explored, with the average level of severity dependent on the shape of a rigid former. Computational studies of these defects can provide information about how they will behave, but it is not possible to confirm that a model satisfactorily describes the mechanics of a defect without experimental studies. For example, findings in this study suggest that residual strains may have a significant effect on the remnant strength of laminates containing fibre waviness, while computational studies often focus only on the orientation of fibres [2,13]. The techniques explored in this paper open new avenues for exploring the behaviour of waviness defects and using the resultant experimental data to refine and validate computational models as well as providing new procedures for detecting and characterizing areas of fibre waviness.

## 5. Conclusion

A method of creating in-plane fibre waviness in composite laminates has been developed and used to produce a large number of specimens at six levels of severity of waviness. This method allows the size, position and severity of the defect to be varied. The manufactured specimens were inspected using two non-destructive techniques and a novel assessment technique based on residual strains. By creating a large batch of specimens, the capabilities of the new residual strain-based inspection technique were explored and it was shown to be capable of both detecting and characterizing waviness defects. Two robust Bayesian linear regression models, capable of predicting the ultimate strength of the specimens in bending, were fitted to the ultrasound and residual strain data. The predictions of the ultimate bending moment based on the residual strain measurements were found to have an uncertainty that was 57% of that for the ultrasound-based predictions. This suggests that residual strains could be used, under certain conditions, to inspect laminates for waviness defects. It also indicates that residual strains appear to have a significant effect on the failure of laminates containing waviness defects and, thus, that incorporating residual strains into computational models of the defect could result in improved simulations of its behaviour. The use of these new techniques, for creating and characterizing in-plane waviness defects, has the potential to enhance our understanding of the influence of fibre-waviness defects on the behaviour of composite structures and to provide detailed experimental data for the development and validation of computational models.

## Data accessibility

The experimental data can be found at the Dryad Digital Repository (http://dx.doi.org/10.5061/dryad.1bv87) [32].
